# The effect of macular carotenoids and omega-3 supplementation on performance in precision sports: the sport nutrition intervention in performance exercise (SNIPE) study

**DOI:** 10.3389/fnut.2026.1892170

**Published:** 2026-09-28

**Authors:** Parimala Sivaperuman, Fraser C. Horn, Warren Roche, James Stringham, Richard Swinbourne, John Nolan

**Affiliations:** 1 Nutrition Research Centre Ireland, School of Health Science, South East Technological University, Waterford, Ireland; 2 High Performance Sport Institute, Sport Excellence Singapore, Singapore, Singapore; 3 College of Optometry, Pacific University, Forest Grove, OR, United States

**Keywords:** antioxidants, eye, nutrition, sport performance, vision

## Abstract

**Background:**

Macular carotenoids improve visual performance in healthy individuals, but these benefits have yet to be tested on athletes. The SNIPE study examined how the macular carotenoids [lutein (L), zeaxanthin (Z), meso-zeaxanthin (MZ)] with omega-3 fatty acids (ω3) impact visual functions and sports performance in precision sports.

**Methods:**

In this double-blind, placebo-controlled trial, 67 athletes and players randomly received either an active supplement containing L, Z, MZ + ω3 (the formulation), or a placebo containing sunflower oil over a 9 month period. Key measurements included blood serum L, Z and MZ concentrations, skin carotenoid score (SCS), visual acuity (VA) and contrast sensitivity (CS), subjective visual function, sleep health (SH), and sport specific performance tests during simulated competitions.

**Results:**

Results showed that the active group, compared to placebo, exhibited statistically significant improvements in L (88%), Z (107%), MZ (0 to 0.226 μmol/L) blood concentrations, SCS (25%), CS at 6 cpd during dim condition (*p* = 0.008, η^2^ = 0.141), and SH in archery (*p* = 0.023, η^2^ = 0.242) and Egame (*p* = 0.041, η^2^ = 0.237). Sports performance also improved for the active group, including total shooting score of 60 shots (*p* = 0.047, η^2^ = 0.081) for shooters and archers, and simple motor time (*p* = 0.018, η^2^ = 0.302) and a suggestive trend toward faster choice reaction time (*p* = 0.054, η^2^ = 0.213) for Egame players, when compared to the placebo group.

**Conclusion:**

The study provides novel evidence that supplementing with carotenoids and omega-3 fatty acids enhances visual function and sports performance in precision sport athletes and players. Further research should attempt to identify the exact mechanisms by which these nutrients are enhancing visual function. These findings highlight the opportunity to add these complementary bioactive nutrients of the eye and brain to athletes' diets to help achieve peak performance in sports.

**Clinical trial registration:**

https://doi.org/10.1186/ISRCTN18761383, ISRCTN18761383.

## 1 Introduction

Athletes engaged in precision sports often experience extended and varying light exposure during training and competition. When light exposure is beyond their adaptive state, it can negatively impact their visual function and overall performance (1, 2). For example, in sports like target shooting and archery, athletes need to identify stationary distant targets accurately, while maintaining stable focus and executing precise shots (3–6). Although these sports do not require responding to changing visual information, their visual demands become more pronounced in outdoor settings with varying weather conditions, significantly impacting visibility and performance (as shown in Figure 1). In another precision sport, Esport, players spend long periods tracking multiple moving objects on electronic digital screens to outmaneuver their opponents. This dynamic visual engagement and prolonged exposure to light and glare from digital devices place considerable stress on their visuomotor abilities, hence impacting athletes' responses to these stimuli (7).

**Figure 1:**
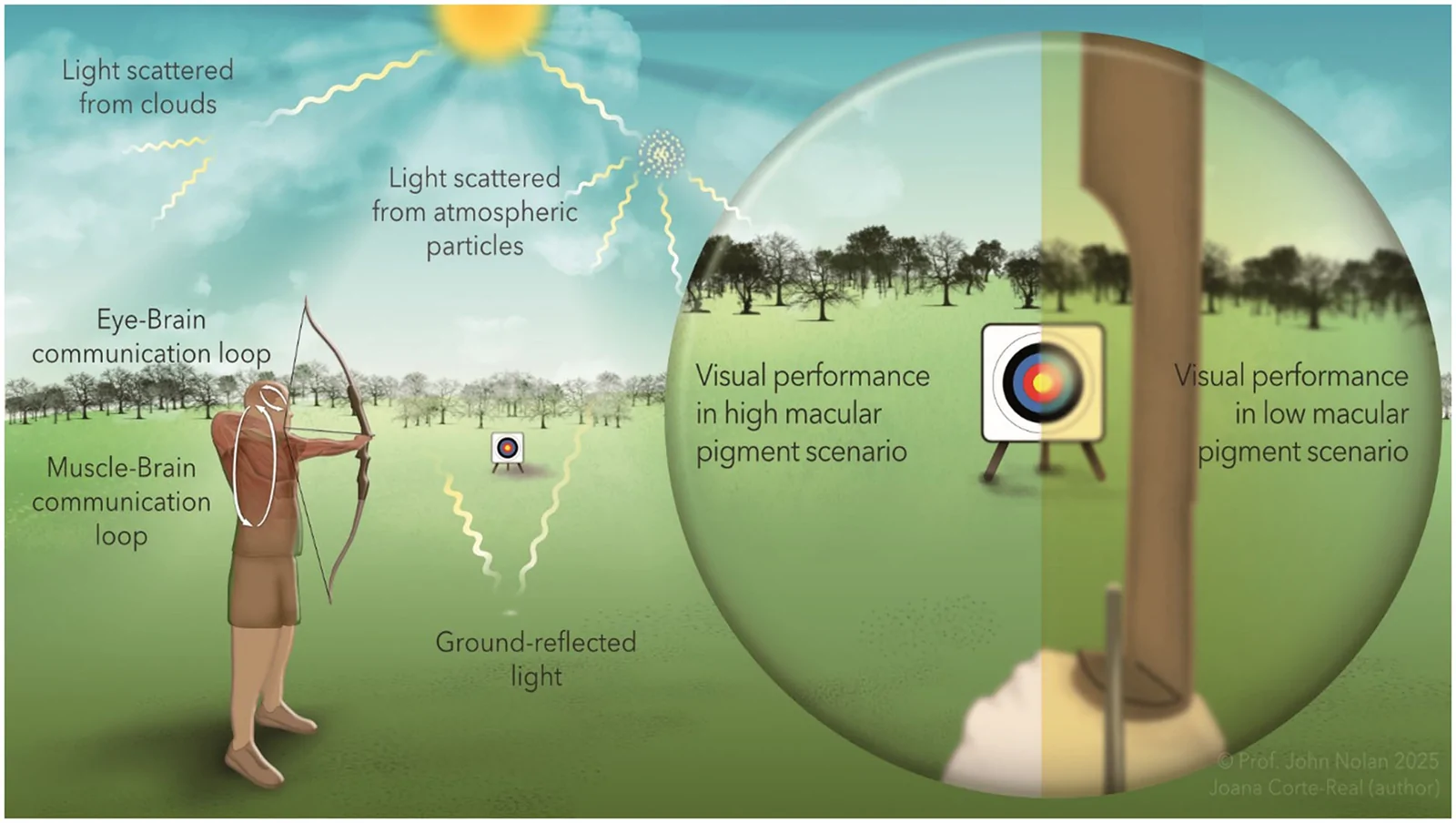
Summarizes how low and enriched macular pigment levels influence an archer's visual performance during outdoor training or competition. The zoomed-in visual image on the right shows an archer with low macular pigment encountering various environmental challenges, such as shadows overlaying cloudy days and glare due to light scattering from sunlight, clouds, and the ground, which significantly diminishes target visibility. This reduction in visibility adversely affects the interactions among the eye, brain, and muscles necessary for accurately aiming the target. Conversely, the left image depicts a hypothesis that with an enhanced macular pigment, the archer can mitigate such challenges effectively by filtering visible blue light and scatter. Figure 1 was generated using Affinity by Canva (https://www.affinity.studio/pt_br/get-affinity) by Joana Corte-Real, PhD, in collaboration with the Nolan Laboratory.

Empirical evidence shows that the human eye is sensitive to exposure to short-wavelength (blue) light (BL), which ranges from 400 and 500 nm in the visible spectrum (8). Visual functions can be affected in two significant ways, which can be summarized via the Health and Optical hypotheses. First, BL can cause photo-oxidative damage to retinal cells, which can compromise eye health (9). Second, the direct effects of BL include issues around light scattering, glare disability and delayed photostress recovery, all of which can degrade or impair visual performance (10–12), including visual acuity, contrast sensitivity (13), and glare discomfort (10, 13). These factors can significantly impact the visual skills of individuals, even those who are highly skilled athletes, in both the short- and long-term. To mitigate the effects of glaring light on their vision, many athletes commonly use visors, caps, tinted glasses or specialized sights (14) and ambient lighting or filters to reduce BL exposure (15) during their sport. While these visual aids offer benefits, they do not fully protect the eyes from photo-oxidative damage caused by the light. Additionally, there are no known studies investigating the impact of daily light exposure incurred outside of training and competition on sport performance.

Nutrition is increasingly recognized as a key component of achieving optimal sporting performances, especially at the elite level (16). Most efforts are directed toward muscle adaptation and recovery, particularly during intense training loads when athletes face chronic muscular and mental fatigue, potentially leading to injury and illness (17). Visual performance in sports is no exception, as healthy eyes are essential for athletes to maintain good vision (15). In our narrative review (18), we discuss the biologically plausible role of the macular carotenoids, specifically lutein (L), zeaxanthin (Z), and meso-zeaxanthin (MZ), in the athlete population. This hypothesis is based on substantial evidence supporting the positive effects of these bioactive nutrients in the general healthy population. These compounds play a vital role in maintaining eye health and enhancing visual function, particularly by protecting the retina from photo-oxidative stress (19, 20). Additionally, recent data from our laboratory has confirmed the impact of carotenoids supplementation on reducing inflammation in the blood (21). The macula, which represents about 4% of the retina area and comprises more than 50% of the visual cortex, provides information on most aspect of high-performance: fine color discrimination, spatial acuity, and motion detection (22, 23). Therefore, enhancing the health of the macula is vital for protecting and improving overall vision.

Omega-3 fatty acids (ω-3) are long-chain polyunsaturated fatty acids that include eicosapentaenoic acid (EPA) and docosahexaenoic acid (DHA) (24). EPA and DHA are stored in the brain and retinal tissue (25). In the retina, ω-3 fatty acids contribute to its structural integrity and functional capabilities (26). Carotenoids are lipid-soluble; their metabolism is strongly linked with lipids. Recent randomized controlled trial studies have demonstrated a significant increase in macular carotenoid concentrations in tissue and serum when participants were supplemented with ω-3 fatty acids (27, 28). Moreover, another study showed that the density of macular pigment in the center of the macula is directly associated with the ω-3 fatty acid index (29). In addition to these functions, certain mediators of ω-3 fatty acids are involved in regulating inflammatory processes and immunomodulation within retinal health (30). These roles are influenced by genetic, metabolic, and behavioral factors that affect DHA tissue status and dietary intake (26). Overall, macular carotenoids and ω-3 fatty acids (EPA + DHA) can accumulate in retinal tissue, highlighting their synergistic role in supporting eye health and function.

Despite this science, there are no robust evidence-based experimental studies that have specifically investigated the effects of dietary and/or supplemented L, Z, and MZ on athletes. Here, we conducted a randomized controlled trial known as the Sport Nutrition Intervention in Performance Exercise (SNIPE) to investigate the impact of supplementation with macular carotenoids (L, Z, MZ) combined with (ω3) (the formulation) on vision and performance in precision sports. Figure 1 illustrates our hypothesis. While our work discusses and describes the role of the carotenoids for eye function, there is a synergistic benefit of the addition of ω3, both from an enhanced bioavailability perspective (31–33) and also from the potential functional role of the ω3 in both the eye and brain (27). Additionally, a secondary outcome of SNIPE was to investigate the impact, if any, of the formulation on sleep health in precision sports.

## 2 Materials and methods

### 2.1 Participants and recruitment

The SNIPE trial recruited Singaporean competitive athletes and players aged between 18 and 50 years from three precision sports, including air-rifle and air-pistol shooting, compound and recurve archery and Egame. Sample size was achieved through convenience sampling registered in their respective sports associations, clubs, and tertiary institutes and the ministry of defense Singapore database. Exclusion criteria for the trial were as follows: consumption of carotenoid supplements in the past 3 months, unable to maintain their current diet throughout the studies, fish and shellfish allergy, presence of ocular disease, color blindness, pancreatic disease, atrophic gastritis, insulin-requiring diabetes, or gastrointestinal bleeding disorders, wheelchair bound and unable to take anthropometric measurements. Each participant above the age of 18 years provided written informed consent of their willingness to participate in the trial. A parent or guardian provided consent for participants under the age of 21, in accordance with the Human Biomedical Research Act 2015 in Singapore.

### 2.2 Study design

This trial was a 1:1 placebo-controlled trial to investigate the impact of supplementation with macular carotenoids (L, Z, MZ) combined with omega-3 fatty acid (ω3; the formulation) on vision and performance in precision sports (Trial registration No. ISRCTN18761383). The trial commenced in September 2023 (i.e., the first subject's visit) and concluded in February 2025 (i.e., the last subject's 9-month visit).

Participants were randomly assigned to either the active or placebo group using a computer-generated sequence from Sealed Envelope Ltd (London, UK; https://www.sealedenvelope.com). Randomization followed a permuted block method with blocks of ten, ensuring balance between groups. The randomization code list was created by a team member who had no involvement in the study design or interaction with the subjects. Participants received supplement bottles only labeled with their identification numbers, and the randomization code was revealed only after the study was completed. The active intervention group received a soft gel capsule with natural carotenoid formulations of L (13.3 mg), MZ (11.7 mg), and Z (1.7 mg); 26.7 mg carotenoids, extracted from marigold petals through patented process (manufactured by the Industry Partner, Industrial Organica), in a fish oil suspension containing 312.3 mg of Docosahexaenoic acid (DHA), 53.5 mg of Docosapentaenoic acid (DPA) and 78.7 mg of Eicosapentaenoic acid (EPA; manufactured by Pharma Marine; the formulation). It is important to note that the choice of carotenoid formulation was informed by over a decade of formulation testing at our laboratory, with a circa 22 mg, 10:10:2 (L:MZ:Z), achieving the best results (33–35). In contrast, the placebo group received a soft gel capsule containing pure sunflower oil. Both groups received their supplements in a sealed container, and the capsules were identical in appearance to maintain blinding. The supplements were third-party tested for ingredient label claims by Supplement Certified and compounds prohibited in sport by Informed Sports. The ingredients, concentration, and formulation have been widely tested in previous clinical trials within our research group and worldwide (28, 33, 36). Participants were required to consume one capsule daily over a 9-month intervention period. Compliance of consuming supplements was monitored throughout the study. The study was approved by the Ethics Research Committees of the High-Performance Sport Institute (Singapore) and South East Technological University (Waterford, Ireland).

### 2.3 Study evaluations

#### 2.3.1 Demographic, lifestyle, medical, ocular assessment

Participant's age, date of birth, sex, ethnicity, education, smoking habits (history and frequency), alcohol intake (average consumption per week, frequency), sports training session (number of sessions per week, duration of each session), years of sports experience, medical and vision histories, which includes ocular treatment or surgery and ocular diseases, were obtained at the start of the intervention.

#### 2.3.2 Anthropometry measurements

Height (cm), weight (kg), 4 skinfolds measurements (triceps, subscapular, biceps and Iliac Crest) and waist circumference were obtained to derive percentage of body fat from all participants.

#### 2.3.3 Sleep health assessment

Sleep health (SH) was evaluated using the validated RU-SATED questionnaire (37), which assessed various aspects of sleep that can affect sport performance. The assessment includes sleep satisfaction, alertness, timing, efficiency, duration, and regularity. The questionnaire comprises polytomous responses on a 3-point Likert scale from 0 (Rarely/ Never) to 2 (Usually/ Always). Higher scores reflect better SH.

#### 2.3.4 Carotenoid assessment

##### 2.3.4.1 Dietary L and Z intake

Lutein and Z dietary intakes over the past 12 months were obtained from a semi-quantitative, self-administered, and validated Dietary Questionnaire for epidemiological studies (38) at the beginning of the study. The questionnaire covered five types of dietary intake (cereal food, sweets and snacks; dairy products, meat and fish; fruits; vegetables; alcoholic beverages), incorporating 80 food items. This questionnaire was conducted in person using an electronic format in front of the researcher to ensure a more efficient collation process and accurate analysis.

##### 2.3.4.2 Skin carotenoid concentration

Total carotenoid concentrations in the skin were measured using optical signals (resonant Raman spectroscopy) from the Nu Skin Pharmanex S3 scanner (Pharmanex^®^ BioPhotonic Scanner), a non-invasive instrument that uses Raman spectroscopy technology (33). By emitting a low-energy blue light beam, this method accurately identifies carotenoids without interference from substances. This technique generates a skin carotenoid score (SCS) by measuring carotenoid concentration in the skin between the maximal and distal palmar creases below the fifth finger of the right hand. Participants align their right hand with the scanner for 30 s to obtain the measurement, which is repeated three times to give a mean score.

##### 2.3.4.3 Blood serum carotenoid (L, Z, and MZ) concentrations

Non-fasting blood samples were collected to monitor the concentration of L, Z, and MZ in participants' blood. Using the standard venepuncture techniques, blood samples were collected in 9 mL blood collection tubes containing a “Z serum Sep Clot Activator.” The tubes were thoroughly mixed for a minimum of 5 min with the clot activator. Blood samples were left at room temperature for 30 min to clot before being centrifuged at 3,000 rpm for 10 min to separate the serum from the whole blood. The serum was transferred to light-resistant microtubes, which were labeled according to the participant's serial number and stored in a Cryovial box at−80 °C freezer. The temperature-controlled sealed cryovial boxes, kept at −70 °C (dry ice), were shipped directly to Supplement Certified Ltd at Howard Biochemistry laboratory to conduct serum carotenoid analysis using high performance liquid chromatography (HPLC), using a method that has been previously described (35).

#### 2.3.5 Visual function assessments at the start and end of the study

##### 2.3.5.1 Visual function questionnaires

Subjective visual function was evaluated using a visual function questionnaire (VFQ), a study-specific questionnaire designed solely to understand participants' perception of their visual functions and their impact on sporting performance. The questionnaire comprises polytomous responses on a 7-point rating scale to evaluate changes in visual response associated with daily and sports tasks. Two questions concerning daily tasks asked participants to rate their overall visual abilities for tasks such as driving and reading street or traffic signs, as well as their abilities to read books and play cards. The sports-related four questions focused on the participants‘ assessment of their visual clarity during shooting or gaming, their comfort level with bright lighting while shooting or gaming, their confidence in visual performance, and their ability to block out distractions and concentrate fully on the shooting or gaming task.

##### 2.3.5.2 Spectacle refraction, visual acuity test, and ocular dominance

Habitual eyewear used, if any, was recorded from verbal assessment. A digital logMAR test chart (M&S Technologies) was used to screen the distance static visual acuity for each eye at a viewing distance of 2.5 meters with their habitual correction (see Figure 2). Using a letter-scoring visual acuity rating, i.e., 20/20 visual acuity assigned to a value of 100, VA was recorded (39).

**Figure 2:**
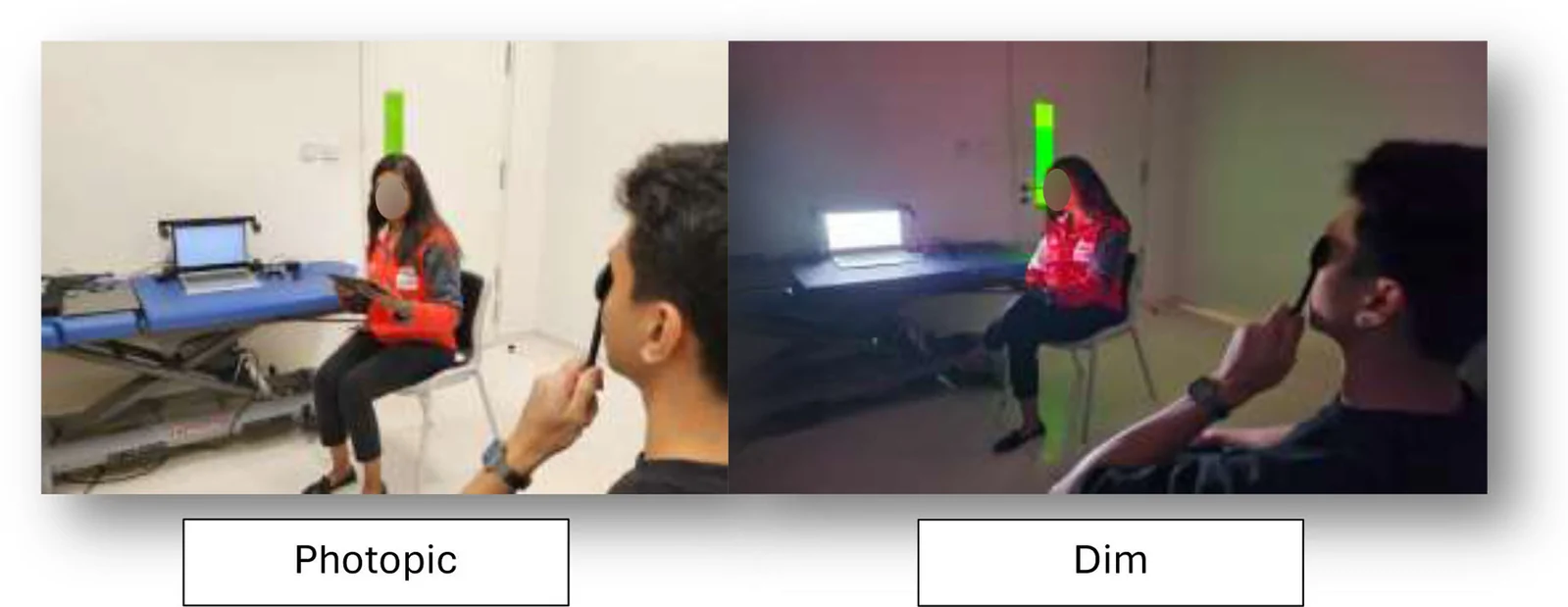
Visual function test using M&S Smart System to assess visual acuity, contrast sensitivity and glare at 6 and 12 cycles per degree (cpd), under photopic and dim conditions for each eye.

To determine ocular dominance, arms were extended in front, and a triangle was formed with thumbs and pointer fingers placed together. To identify the dominant eye, one eye was closed interchangeably, looking through the triangle, formed with thumbs and pointer fingers placed together at arm's length, at a distant object (40). This test also validated the dominant eye for the participants.

##### 2.3.5.3 Contrast and glare sensitivity

The M&S Smart System was used for this study; it is a valid alternative to Pelli-Robson chart. The Smart system uses computer-generated standard Snellen and Sloan Letters presented at contrast levels from 100 to 0.4% contrast. This test identifies true contrast thresholds (41, 42) . The glare testing system, attached to the M&S Smart System, was used to test the existing luminance standards under glare conditions for each participant (see Figure 2). Contrast sensitivity (CS) and glare tests were assessed at 6 and 12 cycles per degree (cpd), with an average luminance 554 lux for photopic (bright) and 21.7 lux for dim conditions for each eye. Participants were given 5 min to adapt to the dim conditions prior to the test.

#### 2.3.6 Sports performance assessment

##### 2.3.6.1 Variables and apparatus

Shooters' performance was evaluated using the optoelectronic SCATT model MX-W2 (SCATT Electronics LLC, Moscow, Russia). This training device contains an optical transmitter-receiver unit that weighs 56 grams and measures 34 × 35 × 60 mm. The software employed for this evaluation was version “Scatt Expert.” The SCATT device was connected to a compatible laptop to facilitate data collection. The archers were evaluated by the Mantis X8 (Mantis Tech, LLC), a compact battery-operated device that combines a precision accelerometer, gyroscope, and magnetometer, was used to record the timing of archers' shots by capturing movement patterns of the bow. This device attaches to the bow's riser and connects to an MantisX application (version 1.7.5) on a tablet or mobile phone via Bluetooth. All Egame players were examined using the Vienna Test System (VTS; Schuhfried GmbH, Modeling, Austria), using an ergonomic response panel while viewing the online tests on a laptop (Microsoft Surface Laptop 5). The SCATT system, Mantis X8, and VTS were used to register variables described in Table 1.

**Table 1:** Descriptions of sports performance variables.

Component	Test: variables (units)	Equipment			Descriptions
		Shooting	Archery	Egame	
Overall performance	Shooting score (points)	SCATT			Shot scores are measured in decimal points, with a range from 0 to 10.9.
			Manual recording		Shot scores are measured without decimal points, with a range from 0 to 10
	Simple reaction time test (SRT)-S1: reaction and motor time (seconds)			VTS	Measures the mean reaction time and the mean motor time upon detecting a visual stimulus, a red light.
	Choice reaction time (CRT)-S4: reaction and Motor Time (seconds)				Measures the mean reaction time and the mean motor time upon detecting visual stimuli, combined red and yellow lights.
	Determination test (DT)-S6: reaction stress tolerance (seconds)				The median reaction time (speed) for pressing a button on the response panel after seeing complex visual stimuli, i.e., red, blue, green, yellow, and white light.
Focus impacting accuracy	Total aiming time (seconds)	SCATT	Mantis X8		The time of the aim point that is continuously located on the target.
	Signal detection test (SDT)-S1: focused attention (counts)			VTS	The total number of correct and delayed reactions to a critical stimulus, i.e., detecting a square with four dots.
Precision	Shots grouping (millimetres)	SCATT			The distance between the center points of a series of recorded shots on a virtual target indicates how tightly clustered the shots are, reflecting the overall pattern of multiple shots.
			Manual calculation		The mode of estimated distances for 60 shots is based on scores, reflecting consistent distances recorded for each shot.

SCATT, scatt system; Mantis, mantis X 8 system; VTS, visual test system; MR, manual recording; MC, manual calculation.

##### 2.3.6.2 Sports performance protocol

Participants were briefed to familiarize themselves with the test situation, which included the assessment structure (i.e., rules and time to complete shots), and trial before each test. To prevent any potential impact on the performance of study participants, consumption of caffeine-containing foods and drinks was restricted for 24 h prior to each assessment. During the sports performance testing, participants were prohibited from using tinted sights, polarized filters, visors, and caps. Ear plugs were used to block all audio cues during the session (43). The performance tests for all sports were administered to each individual around the same time of day before and after the intervention.

Before beginning the actual test, all shooters practiced by taking unlimited trial shots for 15 min with either their own air rifle or air pistol at a target set 10 m away in an indoor range with controlled lighting conditions. According to the shooting system introduced by the International Shooting Sport Federation (44), shooters completed 60 shots, divided into 6 segments of 10 shots each, within a 45-minute duration to simulate competition stress during the session. Shooting participants performed their shots while in a sitting position, utilizing a bench rest to support the arm and a tripod stand to stabilize the weapon, aiming to minimize body and hand movement (45). The shooters' rear aperture sight on all rifles was adjusted to 1.1 to 1.2 mm.

Similarly, archers shot in controlled lighting conditions in an indoor range, but from a distance of 18 m, using compound and recurve bows. Like the shooters, the archers also had unlimited practice shots for 15 min and were required to shoot 60 arrows, divided into six segments of 10 shots each, at a 10-ring color-coded target within a 45-min time frame to simulate a competition session. The archers shot in their usual shooting position (either standing or seated), unsupported. Supporting participants' bows, bodies, and arms, will restrict them from shooting. The SCATT system and Mantis X8 were calibrated during the trial.

All Egame players underwent eye-hand reaction and motor assessments using the Visual Test System (VTS), which consisted of four online computerized tests with a total duration of 45 min (see Table 1). The VTS automatically calibrates the laptop to provide appropriate visual stimuli during the trial. Players were seated at eye level with the computer screen, ensuring their arms rested comfortably on the table to facilitate their responses using an ergonomic response panel. They were required to respond to various visual stimuli as quickly and accurately as possible during the assessments. For tests that required the use of only one hand, athletes and players chose a preferred arm throughout the test to standardize response consistency. Figure 3 illustrates sports performance tests conducted for shooting, archery, and Egame athletes and players.

**Figure 3:**
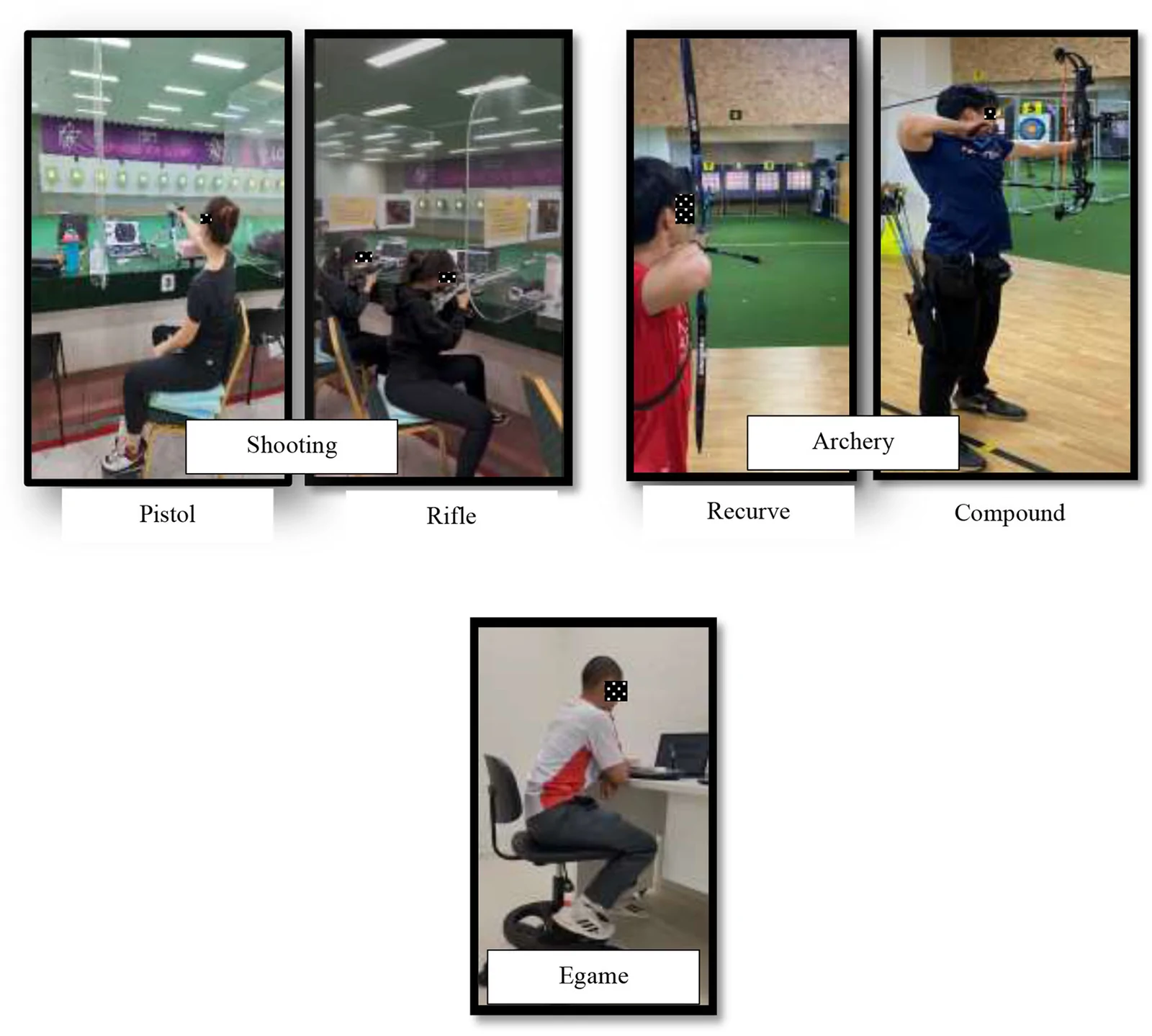
Sport performance test conducted for shooting, archery, and Egame athletes and players.

### 2.4 Statistical analysis

The statistical package IBM SPSS version 28 (International Business Machine Corporation, New orchard Road Armonk, New York, United States of America) was used for all analyses, applying a significance level of 5%. Continuous data were reported as mean (±standard deviation), and effect sizes, where appropriate. Effect sizes are interpreted using Cohen's (1988) conventional benchmarks for partial η^2^: 0.01 (small), 0.06 (medium), and 0.14 (large) (46). An independent sample *t*-test was conducted to compare active and placebo groups at baseline. Data from archery and shooting were standardized for shooting score. Repeated measures of ANOVA analysis was performed for all quantitative variables (i.e., nutritional/biomarker outcomes, shooting/archery performance outcomes, Egame performance outcomes, sleep health outcomes, and visual function outcomes) over time (i.e. over 9 months period) with treatment (active or placebo) as between-subject factor. We also assessed each outcome for any assumption violations. Participants who failed to complete the final follow-up assessment were excluded from the final between-group analysis.

## 3 Results

A total of 72 participants from three precision sports, including shooting, archery and Egame, were enrolled at the beginning of the study, with 67 (93%) participants completing the final assessment at 9 months. Three (4%) participants were lost to follow-up due to loss of contact, and two (3%) discontinued the study due to suspending the sport in the middle of the intervention phase, and another for an unknown reason (Figure 4).

**Figure 4:**
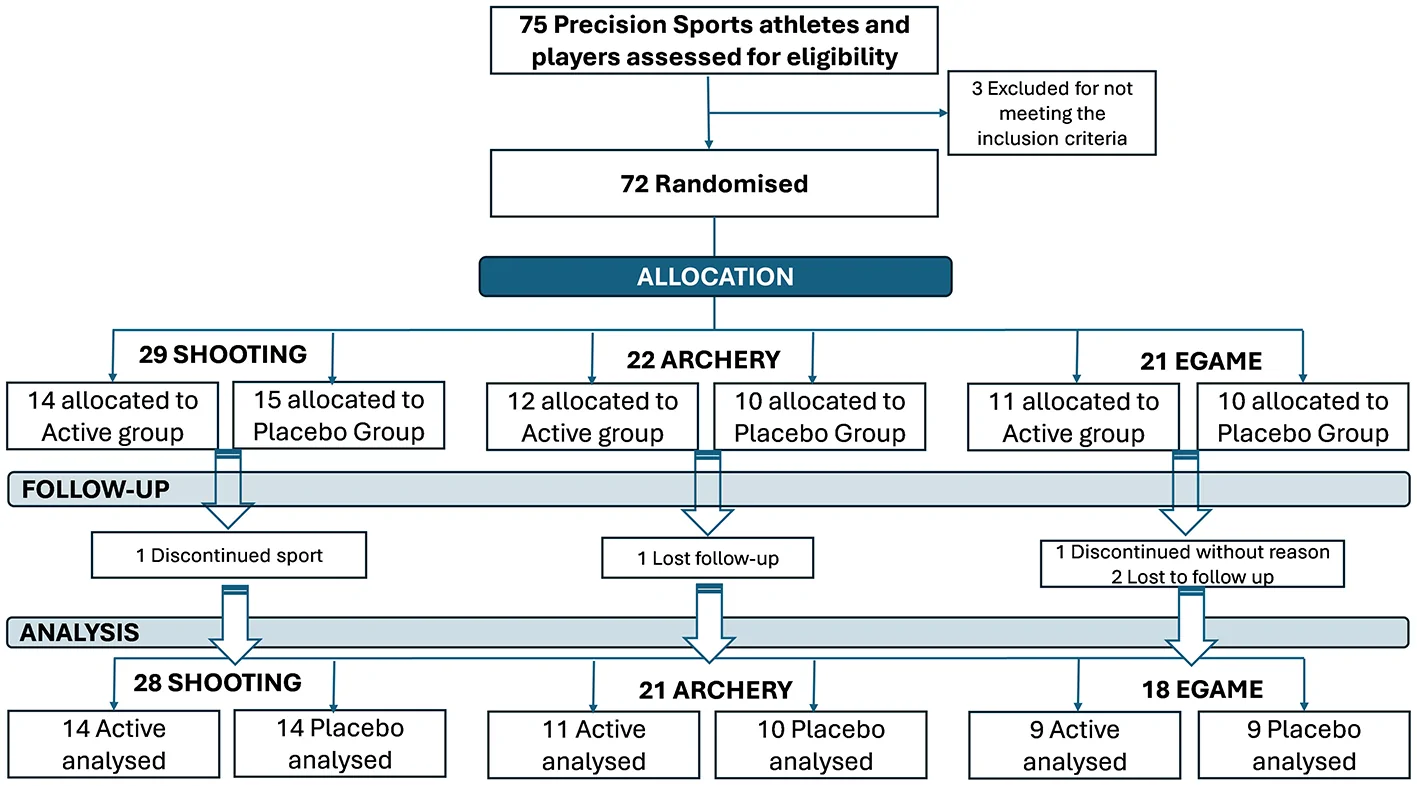
Screening, randomization, and follow-up of study participants.

### 3.1 Baseline findings

Table 2 shows the demographic, health, lifestyle and visual functions data of all three intervention precision sports, including shooting, archery and Egame. The mean (±SD) age of the precision sports athletes and players was 27.53 (±7.32) years, ranging from 18 to 50 years old. Of the participants, 65% (*n* = 47) were men. The largest ethnic group was Chinese, comprising 86% (*n* = 62) of participants, followed by Indian, Others and Malay participants, 7%, 4% and 3%, respectively. The baseline data on education, percentage of body fat, and smoking habits were statistically comparable among the three sport groups. However, there was higher dietary L & Z intake (*p* = 0.05) and a difference in ethnicity (*p* = 0.04) and years of sports experience (*p* = 0.02) between the active and placebo shooting group. Nevertheless, all three sports have a mean L & Z dietary intake value below the recommended intake (6 mg/day) (47) with optimal visual function and a moderate carotenoid score (30,000–49,000) from SCS at the start of the study. Of the nine visual function variables (i.e., VA, CS at 6 and 12 cpd under photopic, dim, and glare conditions in both photopic and dim settings) assessed at baseline, three, including CS 6 cpd under photopic conditions, 12 cpd under dim conditions, and 12 cpd under photopic with glare conditions, showed significant differences (*p* < 0.010, *p* = 0.008, *p* = 0.020, respectively) between the three sport groups. No adjustment was carried out for multiple comparisons.

**Table 2:** Baseline characteristics by intervention groups of three precision sports.

	Precision sports (*n* = 72)			Shooting (*n* = 29)			Archery (*n* = 22)			Egame (*n* = 21)		
Variables	Active, *n* = 37	Placebo, *n* = 35	Sig.	Active, *n* = 14	Placebo, *n* = 15	Sig.	Active, *n* = 12	Placebo, *n* = 10	Sig.	Active, *n* = 11	Placebo, *n* = 10	Sig.
Age (years)	24.95 (±5.79)	30.26 (±7.86)	0.101	24.85 (±6.16)	33.47 (±9.58)	0.169	23.58 (±4.38)	28.50 (±6.62)	0.123	26.55 (±6.71)	27.20 (±3.94)	0.074
Sex, % male	67.6	62.9	0.680	50.0	53.3	0.860	75.0	80.0	0.781	81.8	60.0	0.270
Percentage of Body Fat	21.21 (±7.54)	24.00 (±8.40)	0.374	21.99 (±6.67)	24.44 (±8.82)	0.650	20.07 (±10.06)	21.67 (±10.49)	0.566	21.49 (±3.81)	25.68 (±5.10)	0.149
Diet, L & Z intake (ug/day)	4078.61 (±7143.31)	20558.64 (±1977.96)	0.090	3250.39 (±4402.32)	2917.99 (±1807.79)	0.050^*^	5657.57 (±11605.0)	3252.41 (±2486.37)	0.210	3410.21 (±2432.08)	1325.83 (±1037.05)	0.110
Education, highest level %	97.3	100.0	0.331	100.0	100.0	1.000	100.0	100.0	1.000	90.9	100.00	0.340
Years of Sports Experience	8.98 (±6.03)	10.65 (±6.86)	0.28	9.23 (±2.30)	12.80 (±4.59)	0.02^*^	5.01 (±5.20)	5.75 (±4.61)	0.73	12.01 (±7.63)	12.50 (±9.53)	0.916
Sports Training hours per week	12.32 (±6.74)	12.11 (±8.50)	0.240	15.58 (±7.41)	10.01 (±9.92)	0.940	12.46 (±6.10)	13.50 (±6.69)	0.650	11.44 (±7.36)	10.30 (±6.27)	0.896
Ethnicity, %												
Chinese	83.80	88.6	0.453	64.3	93.3	0.042^*^	91.7	90.0	0.947	100.0	80.0	0.130
Malay	0	5.7		0	6.7		0	10.00		0	10.0	
Indian	8.1	5.7		21.4	0		0	0		0	10.0	
Others	8.1	8.0		14.3	0		8.3	0		0	0	
Smoking, %												
Never smoked	94.6	88.6	0.371	92.2	80.0	0.343	91.7	100.0	0.349	100.0	90.0	0.294
Past smoker	0	2.9		0	6.7		0	0		0	0	
Current smoker	5.4	8.6		7.1	13.3		8.3	0		0	10.0	

Data displayed are mean ± standard deviation (SD) for numerical data and percentages for categorical data; *n*: sample size; Sig.: probability significance *p*-value; %: percentage; ^*^: statistical significant difference observed (*p* < 0.050). Statistical significance was observed between active and placebo for dietary L & Z intake, ethnicity and years of sports experience in shooting groups.

### 3.2 Longitudinal findings

#### 3.2.1 Supplement compliance

Sixty-seven participants were given a total of 270 capsules to consume over a 9-month period. The number of capsules consumed was reported by the participants. The expected number of capsules to be consumed during the study was calculated by multiplying the daily dosage by the number of days since the capsules were dispensed. The percentage of capsule consumed was calculated as follows:


$$\begin{array}{l} \%\text{Capsule consumed =}\\ \frac{\text{Total capsule dispensed}-\text{Number of capsule left}}{\text{Expected number of capsule taken}}\times \text{100}\% \end{array}$$

Seventy percent (70%) of athletes achieved at least 91% compliance by consuming one capsule per day. Only one person reported a poor 50% compliance. Compliance was also assessed at the end of the study by quantifying L, Z, and MZ concentrations in serum, using high performance liquid chromatography (HPLC; see Figure 3). Compliance levels did not influence participant inclusion in the final analysis.

#### 3.2.2 Observed change in nutritional status and body composition

Table 3 summarizes the observed changes in nutritional variables for active and placebo groups following the 9-month intervention period. There was a statistically significant difference with a large effect size between intervention groups over time in SCS and serum L, Z, and MZ levels [*p* < 0.001 for all, η^2^ = 0.264, 0.379, 0.389, and 0.403, respectively (Figure 5)]. Individuals in the active group exhibited notable and significant improvements in the levels of L (88%), Z (107%), MZ (0 to 0.226 μmol/L), and SCS (25%) in comparison to the placebo group in comparison to placebo group. There was no change in body fat percentage in the active and placebo groups. Therefore, body fat percentage is not considered to be a contributing factor to carotenoid concentration over the study period.

**Table 3:** Observed change in nutritional status and body composition over 9 months between intervention groups in precision sports.

Variables	Active intervention					Placebo intervention					Time X group interaction	
	*n*	Baseline	9 months	% Δ	Outcome	*n*	Baseline	9 months	% Δ	Outcome	Sig.	Effect size
SCS	34	3,0852.95 (±10262.28)	3,8539.22 (±1,2725.81)	+25	Improved	33	3,1651.52 (±1,2982.38)	2,7929.29 (±1,2515.69)	−12	Declined	< 0.001^*^	0.264
Serum L (μmol/L)	34	0.278 (±0.089)	0.522 (±0.263)	+88	Improved	32	0.305 (±0.165)	0.277 (±0.126)	−2.8	Declined	< 0.001^*^	0.379
Serum Z (μmol/L)	34	0.217 (±0.044)	0.445 (±0.218)	+105	Improved	32	0.208 (±0.068)	0.201 (±0.056)	−0.7	Declined	< 0.001^*^	0.389
Serum MZ (μmol/L)	34	0.000 (±0.000)	0.226 (±0.194)	–	Improved	32	0.000 (±0.000)	0.000 (±0.000)	0	No change	< 0.001^*^	0.403
Body fat (%)	34	21.21 (±7.54)	20.96 (±8.76)	–	–	33	24.00 (±8.40)	24.39 (±10.24)	–	–	0.421	0.010

Data displayed are mean ± standard deviation (SD) for numerical data and percentages for categorical data; *n*, sample size.; Sig., probability significance value (i.e., *p*-value); % Δ, average percentage change in per subject; ^*^: statistical significant difference observed (*p* < 0.050); SCS, skin carotenoid score; Serum L, lutein; Serum Z, zeaxanthin; Serum MZ, meso-zeaxanthin concentrations are expressed in μmol/L; Body fat expressed in percentage. Interventions are as follows: active group, L (13.3 mg) + MZ (11.7 mg) + Z (1.7 mg) + DHA (312.3 mg), DPA (53.3 mg), and EPA (78.7 mg) and placebo group (sunflower oil) all provided in 1 capsule. Statistical significance was observed between active and placebo for SCS and serum L, Z, and MZ concentrations.

**Figure 5:**
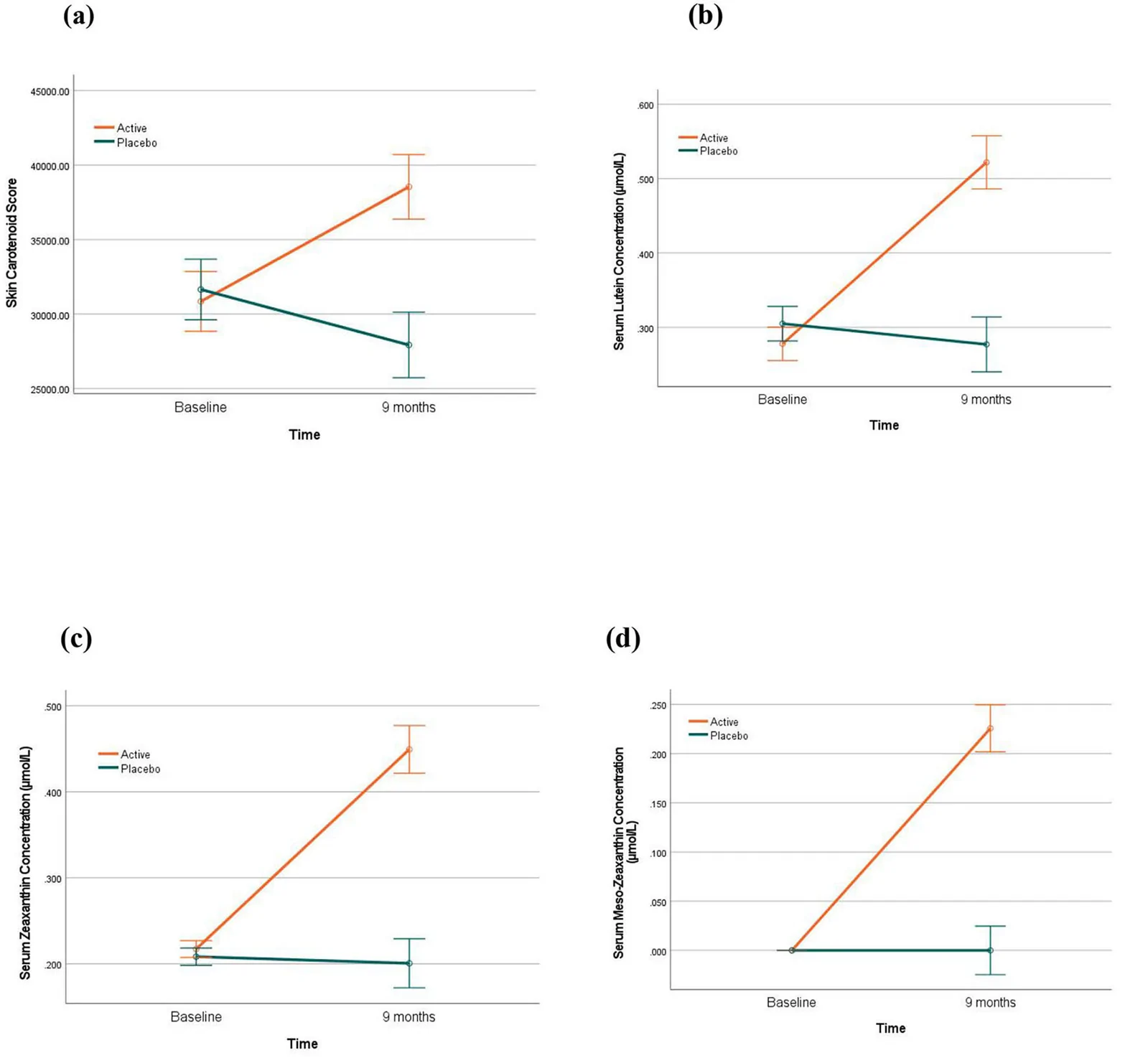
Line charts illustrate change in nutritional status over 9 months between intervention groups in precision sports. **(a)** Skin carotenoid score. **(b)** Serum lutein concentration. **(c)** Serum zeaxanthin concentration. **(d)** Serum meso-zeaxanthin concentration.

#### 3.2.3 Observed change in visual functions

Habitual VA and CS at 6 cpd and 12 cpd frequencies under dim and photopic conditions with and without glare was conducted over 9 months. Data was analyzed using the dominant eyes, which is the shooting eye, of shooters and archers, but for Egamers, both eyes were analyzed based on visual field of play. This study found no significant (*p* > 0.05) change between active and placebo groups for VA in all sports. As illustrated in Figure 6, the mean change in CS at 6 cpd under dim conditions for the dominant eye showed a statistically significant difference between intervention groups with a large effect size (*p* = 0.008, η^2^ = 0.141) between the active and placebo groups for shooting and archery athletes. This statistically significant difference suggests the ability to discriminate letters on the optotype with lower contrast in dimly lit environments, which corresponds to those in the active group identifying letters at a lower percentage of contrast. However, no statistically significant (*p* > 0.05) differences were observed between active and placebo groups for Egamers for any of the visual functions tested. As for subjective visual function assessment obtained via the questionnaire, there were no statistically significant differences (*p* > 0.05) between the active and placebo groups on visual function scores across time between the groups after controlling baseline results.

**Figure 6:**
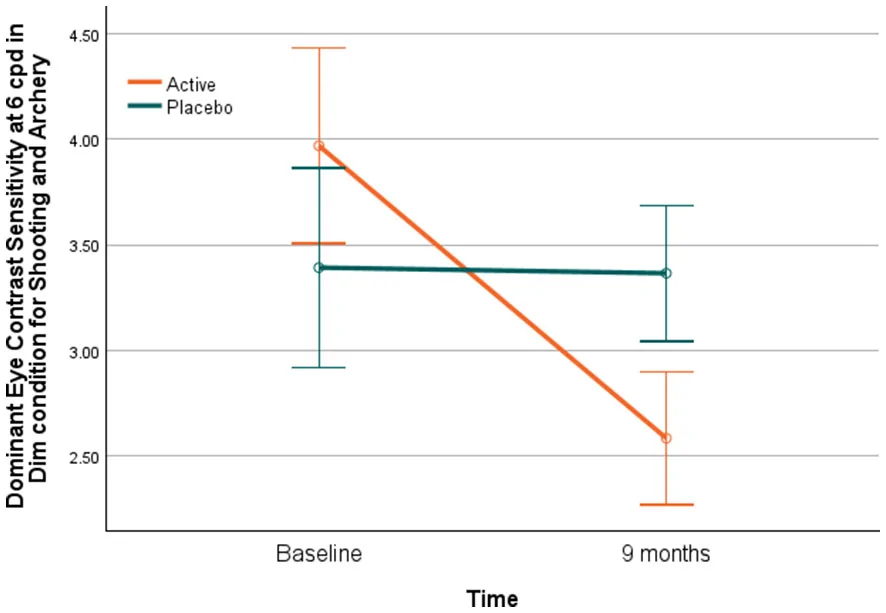
Line chart illustrates change in contrast threshold (%) at 6 cpd in dim condition for dominent eye over 9 months between intervention groups for shooting and archery.

#### 3.2.4 Observed change in sports performance

##### 3.2.4.1 Overall performance

After normalizing the shooting score data from archery and shooting, we observed a statistically significant interaction effect with a medium effect size for the time-over-treatment interaction effect in the score for the total of 60 shots (*p* = 0.047, η^2^ = 0.081), 5th segment of 10 shots (*p* = 0.019, η^2^ = 0.112), and 6th segment of last 10 shots (*p* = 0.046, η^2^ = 0.082) in the active group for shooting and archery when compared to the placebo group (see Figure 7 and Table 4). Individuals in the active group exhibited gradual improvements, compared to baseline, from the 30-shot mark with significant improvement over the 5th segment of 10 shots (2%), the 6th segment of the last 10 shots (2%), and the total 60 shots (3%). In contrast, the score in the placebo group declined after 3rd segment of 10 shots (see Table 4). A statistically significant difference with a large effect size was observed in active for simple motor time (*p* = 0.018, η^2^ = 0.302) and a suggestive trend toward faster choice reaction time (*p* = 0.054, η^2^ = 0.213) for Egame players (refer to Figure 8, Table 5). These significant differences suggest that the active group may demonstrates a faster response speed to the stimuli. No statistically significant difference (*p* = 0.649) was observed for reaction stress tolerance between intervention groups in Egame players.

**Figure 7:**
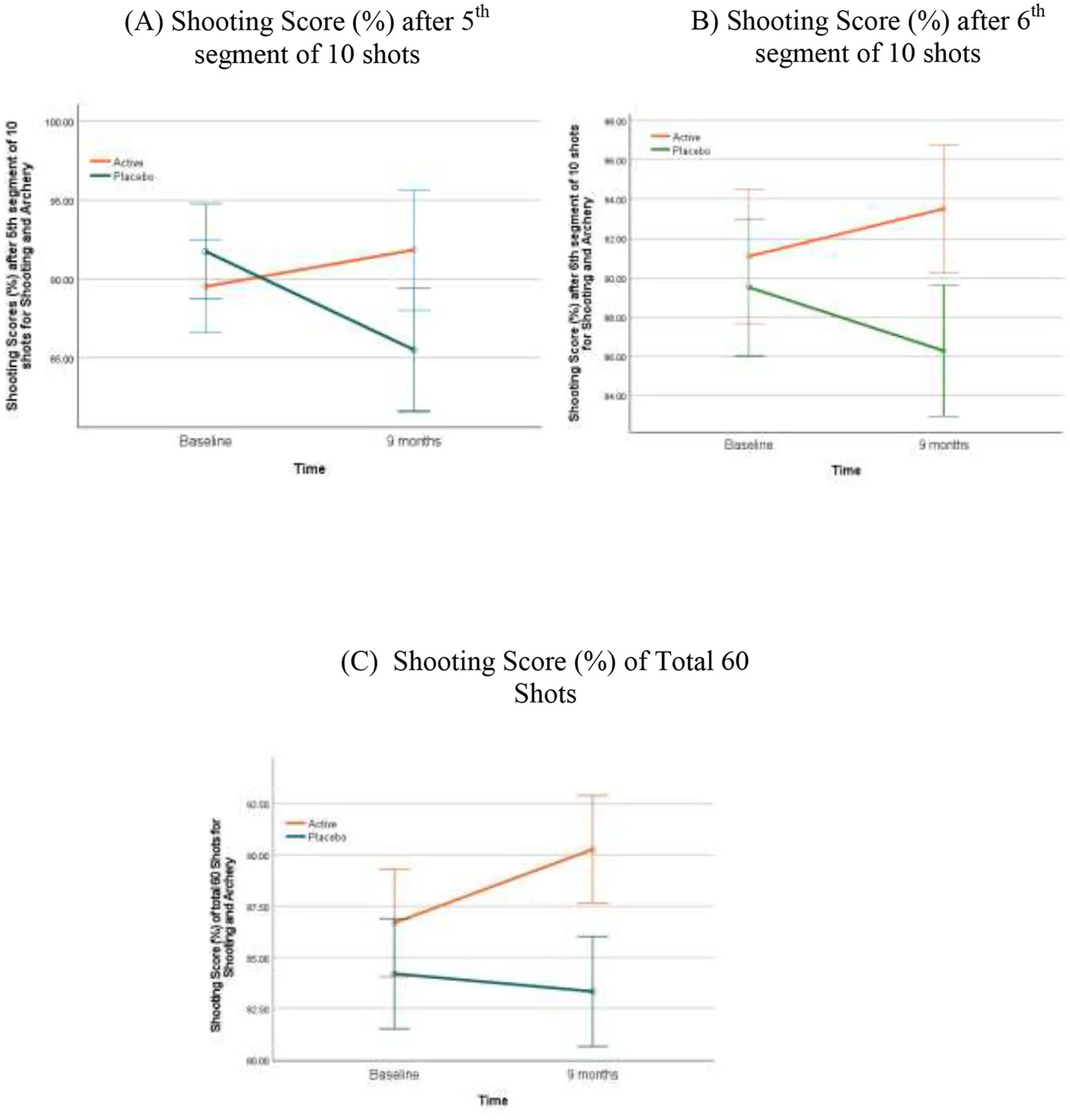
Line chart illustrates change in sport performance over 9 months between intervention groups for shooting and archery. **(A)** Shooting score (%) after 5th segment of 10 shots. **(B)** Shooting score (%) after 6th segment of 10 shots. **(C)** Shooting score (%) of total 60 shots.

**Table 4:** Observed change in sport performance over 9 months between intervention groups for shooting archery.

Component	Variables	Shots	Active intervention (*n* = 25)				Placebo intervention (*n* = 24)				Time X group interaction	
			Baseline	9 months	% Δ	Outcome	Baseline	9 months	% Δ	Outcome	Sig.	Effect size
Overall performance	Shooting score, %	After 10 shots	87.01 (±11.67)	89.45 (±10.19)	+2.44	Improved	82.82 (±18.43)	83.61 (±13.39)	+0.79	Improved	0.525	0.009
		After 20 shots	87.75 (±10.78)	87.22 (±11.58)	−0.53	Declined	81.67 (±18.89)	85.47 (±14.40)	+3.8	Improved	0.225	0.031
		After 30 shots	87.06 (±10.52)	87.20 (±12.97)	+0.14	Improved	85.67 (±15.56)	85.55 (±12.40)	−0.12	Declined	0.939	0.000
		After 40 shots	86.50 (±11.33)	87.94 (±11.32)	+1.44	Improved	83.51 (±16.38)	83.08 (±16.66)	−0.43	Declined	0.451	0.012
		After 50 shots	85.11 (±13.30)	87.17 (±14.46)	+2.06	Improved	86.89 (±11.29)	80.78 (±18.19)	−6.11	Declined	0.019^*^	0.112
		After 60 shots	86.69 (±12.46)	88.87 (±10.41)	+2.18	Improved	84.68 (±17.01)	81.56 (±16.50)	−3.12	Declined	0.046^*^	0.082
		Total 60 shots	86.69 (±10.64)	90.27 (±11.75)	+3.31	Improved	84.21 (±10.64)	83.34 (±14.41)	−0.87	Declined	0.047^*^	0.081
**Component**	**Variable**	**Shots**	Active intervention			Placebo intervention			Time X group interaction	
			***n***	**Baseline**	**9 months**	***n***	**Baseline**	**9 months**	**Sig**.	**Effect size**
Focus impacting accuracy	Total aiming time (s)	Average of 60 shots	25	7.39 (±3.67)	7.61 (±3.07)	23	7.75 (±5.04)	8.33 (±5.19)	0.723	0.003
Precision	Shots grouping (mm)-shooting	Average of 60 shots	14	26.82 (±26.34)	51.84 (±83.14)	14	20.77 (±12.96)	37.06 (±39.55)	0.711	0.005
	Shots grouping (mm)-archery	Average of 60 shots	11	76.36 (±21.57)	87.27 (±34.95)	10	104.00 (±38.64)	108.00 (±56.73)	0.712	0.007

Data displayed are mean ± standard deviation (SD) for numerical data and percentages for categorical data *n*: sample size; %: percentage; sec: seconds; mm: millimetres; Sig.: probability significance *p*-value; % Δ: average percentage change in per subject; Sig: Level of significance of the observed over time between active and placebo intervention groups. ^*^: statistical significant difference observed (*p* < 0.050).

**Figure 8:**
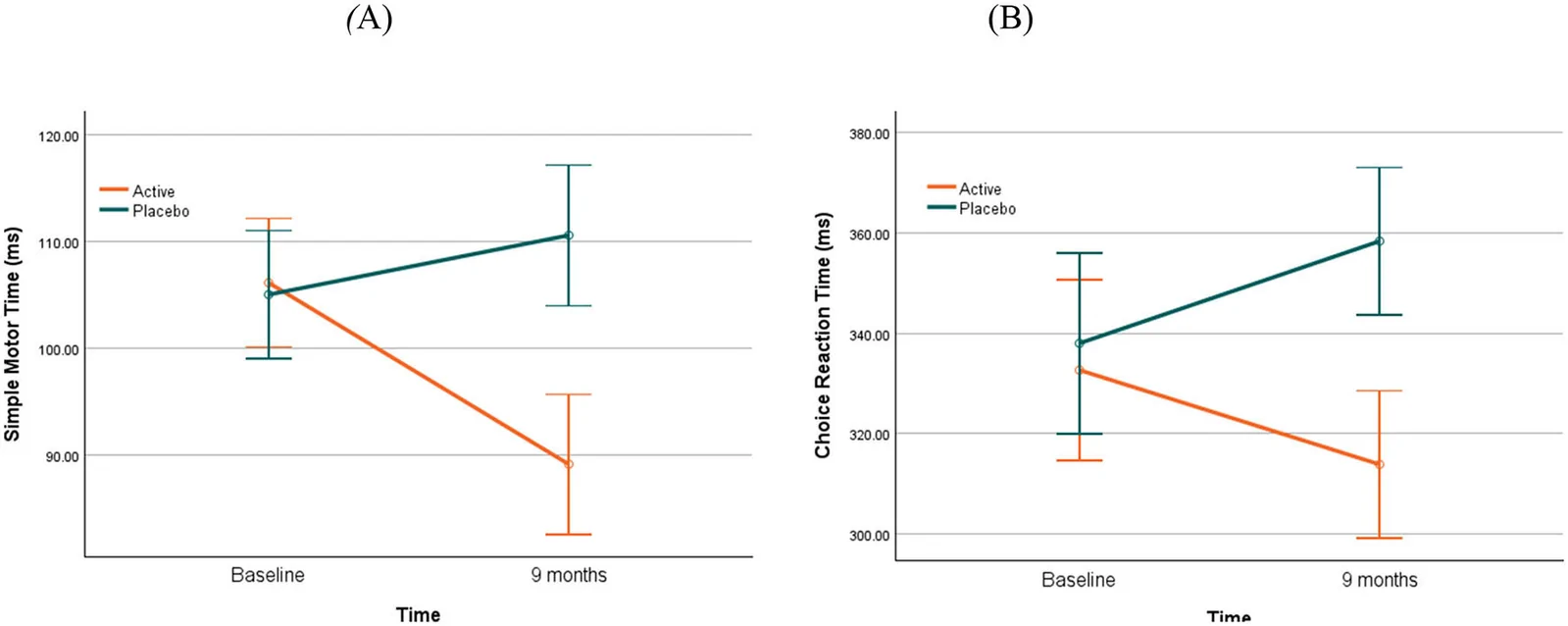
line charts illustrate significant change in sport performance over 9 months between intervention groups for egame. **(A)** Simple motor time (seconds). **(B)** Choice reaction time (seconds).

**Table 5:** Observed Change in Sport Performance over 9 Months between Intervention Groups for Egame.

Component	Variable		Active intervention (*n* = 9)		Placebo intervention (*n* = 9)		Time X group interaction	
			Baseline	9 months	Baseline	9 months	Sig.	Effect size
Overall performance	Simple reaction time (SRT)	Reaction time (ms)	240.56 (±28.0)	248.89 (±33.35)	249.33 (±29.55)	247.89 (±23.44)	0.550	0.023
		Motor time (ms)	106.11 (±17.13)	89.11 (±11.24)	105.00 (±18.76)	110.56 (±25.53)	0.018^*^	0.302
	Choice reaction time (CRT)	Reaction time (ms)	332.67 (±47.40)	313.89 (±37.38)	338.00 (±59.87)	358.33 (±49.74)	0.054^**^	0.213
		Motor time (ms)	105.00 (±18.28)	102.56 (±16.06)	108.67 (±18.73)	113.11 (±26.15)	0.305	0.066
	Determination test (DT)	Reaction stress tolerance (ms)	0.62 (±0.45)	0.58 (±0.09)	0.61 (±0.07)	0.58 (±0.09)	0.649	0.013
Focus impacting accuracy	Signal detection test (SDT)	Focused attention (counts)	52.67 (±5.66)	53.78 (±2.54)	50.78 (±6.50)	53.83 (±3.55)	0.405	0.044

Data displayed are mean ± standard deviation (SD) for numerical data and percentages for categorical data *n*: sample size; ms: milliseconds; Sig.: probability significance *p*-value; Sig: Level of significance of the observed over time between active and placebo intervention groups; ^*^: statistical significant difference observed (*p* < 0.05).^**^Suggestive trend towards statistical significance.

##### 3.2.4.2 Focus impacting accuracy

Total aiming time data cannot be normalized across both sports because of methodological differences in data captured and calculated. No statistically significant difference (*p* = 0.732) was observed for time-over-treatment interaction effect in total aiming time between interventions groups for shooting and archery (refer to Table 4). Similarly, Egame players did not demonstrate any significant difference (*p* = 0.405) in focused attention when comparing the active and placebo groups (see Table 5).

##### 3.2.4.3 Precision

Shots grouping data cannot be normalized across both sports because of methodological differences in data captured and calculated. There was no statistically significant difference in the time-over-treatment interaction effect for shots grouping between the active and placebo groups in shooting (*p* = 0.711) and archery (*p* = 0.712; refer to Table 4).

#### 3.2.5 Observed change in sleep health

The SH data revealed a statistically significant improvement in sleep health score, with a large effect size, for both archery (*p* = 0.023, η^2^ = 0.242) and Egame (*p* = 0.041, η^2^ = 0.237) when comparing the active and placebo groups over time. However, no differences were found between active and placebo groups for shooting (see Figure 9, Table 6).

**Figure 9:**
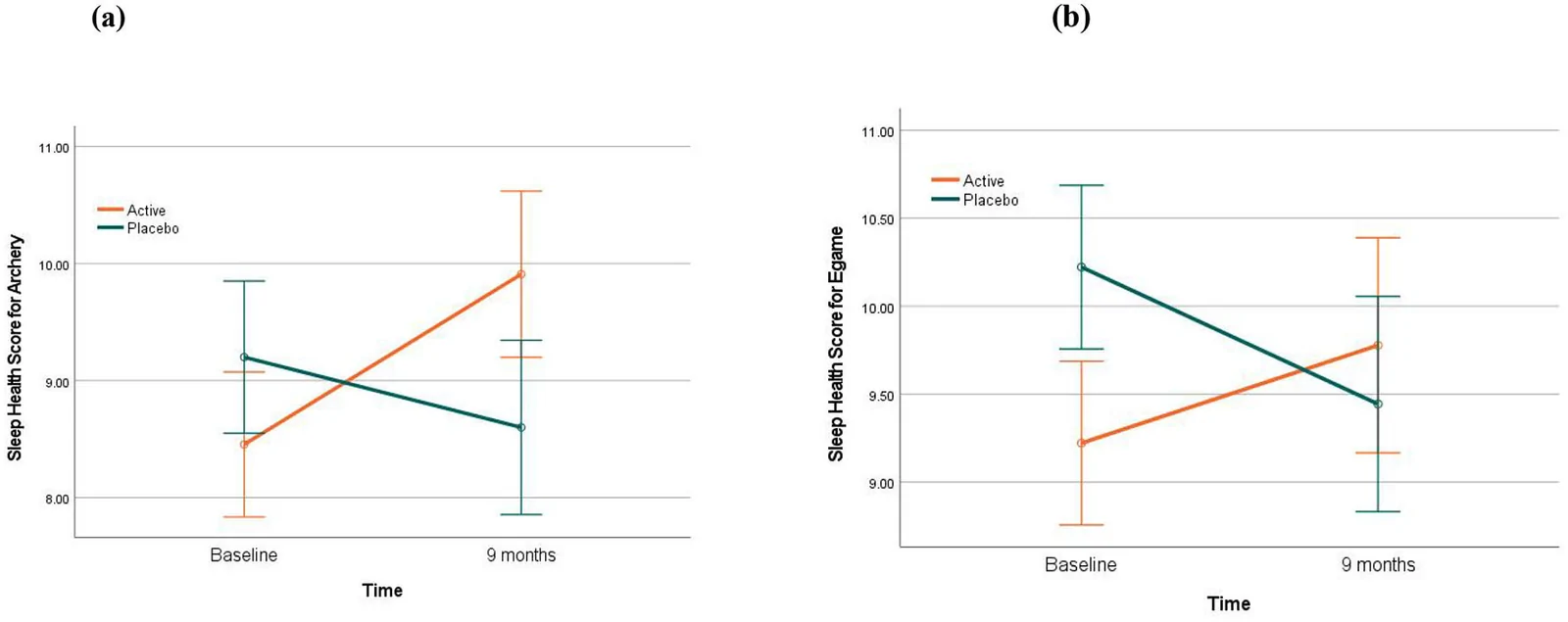
Line charts illustrate change in sleep health scores over 9 months between intervention groups for archery and egame. **(a)** Sleep health score for archery. **(b)** Sleep health score for egame.

**Table 6:** Change in sleep health scores over 9 months between intervention groups for Individual Sports.

Variables	Sports	*n*	Active intervention		*n*	Placebo intervention		Time X group interaction	
			Baseline	9 months		Baseline	9 months	Sig.	Effect size
Sleep health score	Shooting	14	8.50 (±1.51)	9.29 (±2.27)	14	8.64 (±2.98)	9.79 (±1.85)	0.705	0.006
	Archery	11	8.45 (±1.75)	9.91 (±1.70)	10	9.20 (±2.45)	8.60 (±2.91)	0.023^*^	0.242
	Egame	9	9.22 (±1.30)	9.78 (±1.56)	9	10.22 (±1.48)	9.44 (±2.01)	0.041^*^	0.237

Data displayed are mean ± standard deviation (SD) for numerical data and percentages for categorical data *n*, sample size; Sig., probability significance *p*-value; Sig, level of significance of the observed over time between active and placebo intervention groups; ^*^: statistical significant difference observed (*p* < 0.050).

## 4 Discussion

To our knowledge, the SNIPE study is the first trial to investigate the impact of macular pigment enrichment on sports performance in athletes with high dependency on vision and cognitive abilities for their performance. Specifically, this study identified improvements in shooting scores among shooters and archers, as well as in simple motor and choice reaction times among Egamers. This idea was previously discussed by Hammond and colleagues relating to visual performance in baseball players (1, 48), but was not tested using supplement intervention until now. Additionally, the study reports novel improvements in sleep health scores among archers and gamers who were assigned to the active formulation.

### 4.1 Sports performance

In shooting performance, success is measured by the number of points scored on the target. The SNIPE study found that the active group experienced a mean performance improvement of 3% in the shooting score across shooting and archery, whereas the placebo group exhibited an overall decline of 0.87%. Notably, an exponential improvement in shooting score was recorded from the 30-shot mark, with a 2% increase in scores during the last 20 shots leading up to the conclusion of the competition for the active group. In contrast, the placebo group displayed no such improvement. To consistently achieve the highest shooting scores in shorter shooting and archery competition duration, an athlete experiences heightened visual, physical, and psychological stress and fatigue. Despite these conditions, the athletes in the active group demonstrated the ability to improve performance even during the crucial final stages of the competition. This observed performance improvement represents a significant competitive advantage, particularly for elite athletes, when score margins between competitors are extremely narrow. Furthermore, irrespective of the years of experience in sports that influence varying shooting skill levels (such as intermediate, advanced, and elite) as represented in this study, the findings provide strong evidence for the efficacy of the formulation intervention in enhancing shooting performance among these athletes.

The International Shooting Sport Federation (ISSF) recommends an aiming time, which consists of detecting and releasing a shot, of within 4 to 6 s for rifle (49) and 3 to 4 s for pistol (50). This is because aiming for too long can lead to a distorted image, eye fatigue, and mirage effects (51), which can negatively impact shot accuracy. As for archers, research by Keast and Elliott (52) found that recurve archers typically require an aiming time of 2.12 to 5.82 s, while compound archers aim for 2.12 to 8.40 s to achieve a good arrow shot. In our study, no improvement in aiming time was observed, even though the active group experienced an increase in shooting scores. The significance of aiming time as a performance parameter has shown inconsistent results. A study by Ihalainen et al. (53) on national-level air-rifle athletes and a meta-analysis by Sundaram et al. (54) on rifle and pistol shooters found a correlation between aiming time and mean shot scores. In contrast, Ihalainen et al. (55) and Hawkins (56) found weak correlation between aiming time and shooting scores in elite-level air rifle athletes and air pistol shooting, respectively. Similarly, there is no consensus on the relationship between aiming time and quality shot score in archery (52, 57, 58). A tight shot grouping reflects high precision and consistency in technique. The present study found no observed difference in shots groupings between active and placebo groups. While the active group demonstrated improved accuracy, as evidenced by a higher shooting score, there was no correlation with precision, which is shot grouping. The lack of consistency in technique attributed by the varying skill levels of the athletes may have contributed to the finding of no significant precision improvement in our study.

In Egaming, the ability to respond accurately to different visual stimuli is essential. However, the most critical aspect of this sport for winning is the speed of these reactions (59). Reaction time (RT) refers to the time interval between a visual, auditory or somatosensory stimulus and the initiation of a voluntary response (60). The subtypes of RT are simple reaction time (SRT), which involves one stimulus, and choice reaction time (CRT), which involves multiple stimuli. Interestingly, players in this study demonstrated faster reaction and motor times to visual stimuli than those in Bickmann et al.'s (61) study, who conducted similar SRT and CRT baseline assessments in professional Esports players. A randomized controlled trial by Yoshida and colleague examined the effects of astaxanthin, L, and Z on visual function after the use of a visual display terminal (VDTs), including computers, smartphones, tablets devices and video games consoles, in healthy adults. The results showed that the group receiving active formulation experienced a slower decline in eye-hand coordination after using VDTs (62). When comparing responses between the intervention groups, our study found that the active group exhibited a significantly shorter motor time (referring to processing speed and actions) measured by the SRT and a suggestive trend of quicker reaction times (referring to perceiving a stimulus and initiating a corresponding reaction) for the CRT. Notably, although the difference was not statistically significant, the active group showed a 2.44-ms improvement in motor time on the CRT, representing a medium effect size. Conversely, the placebo group showed a decline in speed in both the SRT and CRT, even though performance was unaffected by stress or focused attention, as evidenced by the reactive stress tolerance and signal detection tests. In sports where quick responses can determine victory or defeat, our current findings indicate that the macular carotenoids have a positive influence on reaction and motor times, particularly under a multiple-stimuli challenge such as CRT. Interestingly, work by Bovier and colleagues demonstrated that L and Z supplement intervention had an impact on visual processing speed in healthy adults (63). This may explain at least part of our findings. Although the exact mechanism is unclear, these nutrient compounds may enhance gap junction communication between neurons and glial cells, potentially improving processing speed (64). Importantly, the results of our study are consistent with this assertion, as they demonstrated L, Z and MZ supplementation enhances the reaction and motor times, i.e., speed of the eye-hand coordination, by boosting visual processing speed.

### 4.2 Visual function

The relative size and contrast of the elements present in this study influence our ability to perceive finer details in a visual scene. An individual's ability to identify the smallest details in a black and white image with 100% contrast is known as visual acuity (VA). Numerous studies in sports have shown that athletes have demonstrated better visual acuity [20/15 (65–68) or better, 20/9.2 (69)] than nonathletes. Specifically in precision sports, Abernethy and Neal (70) cited Lander and colleagues study (71) that aimed to evaluate the skilled performance of elite and non-elite air rifle, air pistol, small bore rifle, trap and skeet shooters. They found that VA in the dominant eye at a short distance (10 m) is one of the important visual characteristics of a successful shooter, as it allows their ability to coordinate visual oculomotor skills better (72). Additionally, a study examining the VA without vision correction of Olympic pistol and rifle shooters agrees with this finding (73), indicating that shooters displayed better binocular VA (elite mean = 1.34 ± 0.26 or −0.1 LogMar equivalent, non-elite mean = 1.54 ± 0.24 or −0.1 LogMar equivalent) than non-athletes (mean = 1.07 ± 0.15 or 0.0 LogMar equivalent), despite having significantly older age athletes, above 29 years of age, than the non-athlete group. In other limited studies focusing on visual acuity in target-based sports, Laby et al. discovered that Olympic-level archers showed superior VA (−0.20 ± 0.12 LogMar or 20/12.5 equivalent) with vision correction when compared to athletes from seven other Olympic sports (65). In our study, athletes were screened for VA of 20/24.06 or more. Although we found no changes in VA in precision sports across both intervention groups, our findings revealed that 94% of athletes involved in shooting and archery had a minimum VA of 20/24.06. Similarly, 95% of the Egame players had a minimum VA of 20/24.06. A comparable result was reported in Yee's study, which indicated that the binocular static VA of video game players (mean = −0.14 ± 0.2 LogMar) was not significantly different from that of other athletes (mean = −0.16 ± 0.01 LogMar) and the control group (mean = −0.13 ± 0.02 LogMar) (74). Notably, in our study, the VA in the dominant eye of precision sport athletes and players was good, despite being older than 29 years of age. One limitation is that some of the studies mentioned employ different or unclear testing protocols for measuring VA, which may or may not account for corrected vision. In contrast, the current study tested the VA of all participants with their habitual correction used for sport, if any. This suggests that participants in this study may fall within the same range if VA threshold test was conducted as those in the previously mentioned studies.

While visual acuity is an important aspect of visual performance, recent studies and reviews have challenged the notion that it is the only measure that matters. They emphasize that beyond acuity, visual memory (75), contrast sensitivity (76, 77), and reaction time (70, 78), fixation (77, 79–82), ocular dominance (83) and eye-hand coordination (75), are important measurable visual functions categories that must not be underrated for peak sporting performance. Interestingly, the results of this current study demonstrate that CS, defined as the ability to detect slight increment in shades of gray against a uniform background (84), is a key determinant of visual performance. However, the active group shooters and archers showed statistically significant improvement in CS at 6 cp in dim conditions. Given that the skin carotenoid concentration and blood serum L, Z, MZ concentration increased in the active group over the study period, it is reasonable to conclude that the improvement in CS is linked to enhanced carotenoid status. These findings align with the CREST study, in which Nolan and colleagues reported significant improvement in CS at 6 cpd in mesopic conditions with increased MP levels, following L, Z, and MZ supplementation in a healthy population (34). In contrast, a recent retrospective study reported that sport motorcycle test pilots experienced improved VA and CS under photopic conditions after taking a food supplement of lutein (6 mg), cyanidin-3-glucoside (20 mg), verbascoside (2 mg), and Zinc (5 mg) for 70 days (85). Good CS is said to be essential for outdoor shooting and archery. It helps athletes to distinguish orange-colored targets against ground background, such as green or brown grass, foliage (trees), and sky (blue, cloudy, or heavy cast sky). One possible mechanism contributing to this capability is the filtration of blue light in the cornea and lens, which helps to reduce chromatic aberration and also reduces the impact of light scatter (34). Improvement in CS in dim condition will be advantageous for navigating the rapidly changing light environment for the majority of shooters and archers who train and compete outdoors in this study. This skill is crucial even in athletes with well-developed cognitive processing of visual information (13, 86). It should be noted that no changes in visual function were observed for the Egame sport. A small sample size may have contributed to lack of statistically significant changes in this sport.

It is also worth noting that the visual assessment used in this study is consistent with the visual characteristics outlined in “The Vision Pyramid,” which was proposed by Kirschen and Laby (87). The details are provided in our narrative review (publication in press). Our findings identify another opportunity for athletes to focus on beyond nutrition. We believe that athletes should include visual function assessment as part of their routine performance measurements.

Lastly, subjective evaluations of visual function showed no changes in any of the participants involved in precision sport. This suggests that the improvements in sports performance for shooting and archery athletes were not influenced by their beliefs about experiencing better visual function. Instead, the enhancements in their actual CS likely contributed to the observed improvements in performance. It's also possible that the active group adapted to the performance improvements during the intervention period, which may have led them to perceive less of a difference subjectively.

### 4.3 Sleep health

Sleep is essential for athletic health, recovery and performance (88). Sleep health encompasses a holistic view of sleep, which includes various domains of sleep characteristics, such as irregularity, alertness during waking hours, timing, efficiency, duration and overall satisfaction, rather than focusing solely on individual sleep symptoms and disorders (89). In the realm of sports, insufficient sleep can impair cognitive performance affecting attention (90), psychomotor skills (91), as well as specific sport-related accuracy (92). Additionally, lack of sleep can negatively impact mental state (93, 94) and hinder immune and endocrine functions that are essential for recovery from training (95). Athletes often endure demanding training and competition conditions, characterized by rigorous training, competition schedules, selection pressures, injury risks, societal stress, international travels and exposure to noise and light, which can contribute to insufficient sleep (96, 97). Furthermore, research indicates that prolonged exposure to BL from electronic devices such as smartphones, tablets, laptops, and televisions for more than 6 h per day can delay the release of melatonin and perturb sleep. This interference can increase alertness and reset the body's circadian rhythm in humans as well (98, 99). Sleep disturbance can disrupt the retinal circadian clock (100). The retina's circadian clock helps the eye adapt to photopic (light) and scotopic (dark) conditions, which correspond with the solar day and night. Dysfunction in this system is linked to affect ocular health and various eye diseases, such as ocular discomfort, blepharospasm, NAION, diabetic macular edema and other retinal vasculopathies (100). Moreover, compromised sleep health has downstream negative effects on cognitive and circadian elements important to performance (98, 99).

A robust baseline sleep health score was observed in our study cohort. To date, only one known study has evaluated sleep and sleep health using the RU-SATED questionnaire on elite sailing and athletic athletes supplemented with kiwi. That study also found good sleep health score at baseline but no observed changes after the intervention (101). In contrast, our research observed positive results for sleep health in the active groups among archery and Egame sports, following the intervention. Our study is the first study to evaluate the impact of L, Z and MZ intervention on sleep health in Egame players and archery athletes. Other studies have documented similar improvements in sleep quality among healthy individuals taking L and Z or L, Z, and MZ supplements, especially those exposed to SBL from digital screen (102–104). Notably, our study also observed positive effects for both sports, despite light exposure for them were noticeably shorter duration, i.e., outdoor training or digital screen for an average 3 h or 2 h per day, respectively. Similarly, research conducted by Stringham and colleagues, which involved a randomized controlled trial using L, Z, and MZ in sunflower oil supplementation among healthy participants, found that sleep quality significantly improved in the active group, which consisted of individuals exposed to digital screens for at least 6 h a day (105). The evidence from our study suggests that the active formulation offers substantial benefits for sleep health, even with limited daily light exposure. Interestingly, a similar improvement was not observed among shooters, as a large proportion of these athletes were traveling for competition abroad during the study period, which may have disrupted their sleep cycle. Stringham and colleagues also noted that the positive impact of macular carotenoid supplementation on sleep may not be the result of altering melatonin secretion from the pineal gland (which affects somnolence), but rather through systemic reduction in oxidation and acting as an anti-inflammatory (105). This could also help explain the results of the present study, which suggest that the active formulation improves sleep health in athletes.

## 5 Strength and limitations

The strength of this double-blind placebo-controlled study lies in its methodology, namely the applied data collection conditions and the elite participants represented in the study cohort during a simulated competition. While a previous study on air rifle shooting (55) found that performance decreases in simulated competitions compared to traditional training. Both training and competition performances are associated with the same technical skills while applying more pressure to the subject. Therefore, using a simulated competition in this study was advantageous, reflecting a competition performance and potentially increasing the value of findings, and should be prioritized in shooting research. Another major strength of this study is its design. The SNIPE trial was a double-blind, randomized clinical trial, meaning that neither the participants nor researchers knew which individuals were consuming the active formulation and which participants were consuming the placebo, where treatments groups were also randomized. Furthermore, SNIPE provided a unique opportunity to investigate whether observable changes induced by supplementation in SCS and S-L, Z, MZ levels were related to observable changes in sport performance among athletes with healthy eyes with low SCS and S-L, Z, MZ level (and therefore, putatively low MP concentrations of L, Z and MZ). The study also offers a non-invasive assessment method to evaluate L, Z, and MZ levels, demonstrating a clear correlation between SCS and S-L, Z, MZ. Such a non-invasive method may be beneficial for sport dietitians, healthcare providers, and nutrition professionals when assessing athletes' macular carotenoids status.

With respect to study limitations. The Egame sport performance test was conducted in a laboratory setting, which may not accurately represent real-time competition situations for Egamers. Additionally, the Egame performance testing was standardized for all players, regardless of the games they played. Future research should involve sport-specific performance tests to a larger sample size to enhance the validity of the findings. This study highlighted improvement in dim CS, which may be advantageous for navigating the rapidly changing light conditions typically encountered at an outdoor shooting range. This finding is consistent with the hypothesis presented by our study, which suggests that light exposure in outdoor shooting environments may negatively influence visual function. However, it is important to note that for our research trial the athletes were tested in an indoor environment. This allows for controlled light exposure during testing, but it is a limitation of our study because for some competition athletes will compete outdoors, which presents different environmental challenges as seen in Figure 1. To build on this research, future studies may explore the effects of varied light conditions in outdoor settings to further examine the impact of light on sport performance. We also recognize that the enhancement of visual functions in sports performance is likely attributable to the formulation used. However, further research is necessary to establish whether the improvement is solely the result of carotenoids, omega-3 fatty acids, or a combination of both. In addition, the findings from this study should be interpreted in the context of the multiple outcomes examined. Nevertheless, future studies to include adequately powered sample sizes and mixed-effect modeling are warranted. We also recommend that a future, larger single-sport confirmatory trial be designed with *a priori* power calculation on the primary endpoint most relevant to that sport to be conducted, as this would be a valuable next step in our research.

## 6 Conclusion

In sports, where winning and losing can be determined by small margins, the findings from the SNIPE study offer opportunities to positively impact on the outcomes relating to sport performance. The implications of the SNIPE study are significant, as it highlights the importance of complementary bioactive nutrients, specifically carotenoids and omega-3 fatty acids, that support optimal function of the eye and brain and, subsequently, positively impacts areas of sport performance. This data represents an evolution in dietary recommendations, enabling stakeholders in the sports community, including sports dietitians or nutritionists, sports scientists, healthcare providers, coaches, and athletes, the opportunity to incorporate these functional nutritional bioactives as part of nutrition management.

## Data Availability

The original contributions presented in the study are included in the article/supplementary material, further inquiries can be directed to the corresponding author.
